# Promising Perinatal Outcome after Using a Simplified Low-Cost IVF Culture System Specifically Designed for Resource-Poor Countries

**DOI:** 10.3390/jcm12062264

**Published:** 2023-03-15

**Authors:** Willem Ombelet, Jonathan Van Blerkom, Liesbeth Bruckers, Nathalie Dhont, Geeta Nargund, Rudi Campo

**Affiliations:** 1 Department of Obstetrics and Gynaecology, Genk Institute for Fertility Technology, ZOL Hospitals, 3600 Genk, Belgium; 2 Faculty of Medicine and Life Sciences, Hasselt University, Agoralaan, 3590 Diepenbeek, Belgium; 3 Department of Molecular, Cellular and Developmental Biology, University of Colorado, Boulder, CO 80309, USA; 4 BioStat, Data Science Institute, Hasselt University, 3500 Hasselt, Belgium; 5 St. Georges Hospital& Medical School, London SW17 0QT, UK; 6 Life Expert Centre, Schipvaartstraat 4, 3000 Leuven, Belgium

**Keywords:** IVF, assisted reproduction, developing countries, infertility care, LMIC, low-cost IVF, simplified IVF

## Abstract

Background: Assisted reproductive techniques services are often not accessible to the majority of infertile couples in Low and Middle Income Countries (LMIC) due to high costs. Lowering IVF laboratory costs is a crucial step to make IVF affordable for a larger part of the world population. We developed a simplified culture system (SCS) which has proven to be effective, and the next step is to prove its safety.Methods: Preterm birth (PTB) and low birthweight (LBW) of 176 singletons born after using the SCS, 105 after fresh embryo transfer (fresh ET), and 71 after frozen embryo transfer (frozen ET) were compared with all IVF/ICSI singletons born in Belgium between 2013 and 2018. When comparing our 105 SCS babies born after fresh ET with all Belgian babies born after conventional IVF only, we also adjusted for 7 risk factors known to influence perinatal outcome, namelythe mother’s age, day of transfer, pituitary inhibition protocol, rank of cycles, number of oocytes retrieved, number of embryos transferred, and gender of the baby.Findings: Before adjustment, we found a significantly higher PTB (10.2% vs. 3.8%, OR 2.852, 95% CI [1.042–7.803], *p*-value 0.0413) and LBW (9.8% vs. 2.9%, OR 3.692, 95% CI [1.163–11.721], *p*-value 0.0267) in the conventional IVF group versus SCS after fresh ET. After adjusting for seven risk parameters, these differences remained significant (PTB: OR 2.627, 95% CI [1.013–6.816], *p*-value 0.0471) and LBW: OR 3.267, 95% CI [1.118–9.549], *p*-value 0.0305). PTB and LBW between both groups was not significantly different for singletons born after frozen ET. Interpretation: Taking into account the small series, PTB and LBW rates in SCS singletons in FRET cycles are very reassuring and significantly lower compared to babies born after conventional IVF in Belgium. Being aware of its effectiveness, our results offer a good perspective for SCS to become an important tool to implement low-cost IVF in LMIC.

## 1. Introduction

Infertility and involuntary childlessness impact millions of women and men. Depending on which criteria are used, the number varies between 52.6 and 200 million couples which are involuntary childless, with most residing in low and middleincome countries (LMIC) [1,2,3]. The overall prevalence of infertility is estimated at 3.5–16.7% in LMIC [4]. The consequences are often devastating: sexual and reproductive health problems, stigma and ostracization, psychological distress, marital instability, gender-based violence, and economic hardship [5,6,7,8]. Infertility in LMIC is mostly caused by severe male infertility and female infertility due to tubal block as a result of sexually transmitted diseases or pregnancy-related infections. In both circumstances, IVF-related procedures are the treatment of choice. Sad enough, in the majority of LMIC these methods are either unavailable or too costly because infertility is insufficiently integrated in sexual and reproductive health policies and there is hardly any funding for infertility. Even diagnosis and low-tech treatment options are not offered systematically at primary healthcare level and are rarely part of national insurance schemes. As a result of this, access to fertility care is subjected to devastating inequities in that mostly only the rich can afford expensive diagnosis and treatments (in the private sector) [5,6,7,8]. Lower cost variations of Assisted Reproductive Technologies (ART) exist, but are not widely introduced.

Since the birth of Louise Brown almost 45 years ago, over 9 million IVF/ICSI babies have beenborn. Unfortunately, due to the limited access to ART in resource-poor countries, this cannot be regarded as a success story since the majority of the world population cannot benefit fromthese new technologies. To increase access and affordability, we urgently need more simplified and cheaper IVF procedures. According to a recent qualitative evidence-based assessment, thekey barriers to the inclusion of fertility care in reproductive health policies in LMIC were (i) the insufficient interest of politicians and healthcare providers, (ii) the failure to adequately recognize the burden of infertility, and (iii) high costs associated with ART [9]. Recent WHO studies and statements clearly indicate an urgent need to improve the availability, quality, and accessibility of ART worldwide. This is especially evident in LMIC where the high cost of ART treatments, where available, have devastating consequences, which have resulted in considerable interest of the WHO in developing affordable IVF procedures [8,10].

Increasing global access to infertility care including lowering the costs associated with IVF laboratory procedures was the first aim of the Walking Egg project [11]. Consequently, a novel method for IVF was introduced, called the simplified culture system (SCS) [12]. By using the SCS, we minimized the complexity of the conventional IVF laboratory by avoiding the requirements for medical grade gases, ‘high-tech’ incubation equipment, and expensive infrastructure we usually find this in high resource settings.

In SCS, we only need 2000–5000 motile-washed spermatozoa for the insemination of oocytes, which makes this system feasible for use if a moderate or severe male factor is involved [12].

Many problems frequently occurring in regular IVF laboratories, such as unwanted temperature changes, in-room air quality problems, impurities in gas supply, etc., can be avoided because development from insemination to transfer is undisturbed in the same tube until embryo transfer. Another benefit of this closed culture system is that it affords a relatively large atmospheric reserve, which owing to a greater surface area for gaseous diffusionmaintains appropriate equilibration in larger volumes of bicarbonate-based media favoured for clinical IVF and preimplantation embryogenesis; this system precludespotential problems that can occur when small, microliter drops culture is performed under oil for prolonged periods such as changes in pH or composition (e.g., accumulation of ammonia) that are biproducts of oxidative metabolism by living sperm, residual cumulus/corona cells and the preimplantation embryo, or embryos whencultured in groups.

We recently reported on the effectiveness of this method when compared to ICSI, followed by regular culturing using sibling oocytes in a selected population of non-severe male infertility. We found no difference in ongoing pregnancy rate, implantation rate, and miscarriage rate between SCS and ICSI [13].

With respect to safety for the SCS, perinatal outcome data are of utmost importance. Pregnancies following IVF/ICSI, when compared to naturally conceived pregnancies, are more likely to be affected by perinatal complications such as preterm birth (PTB, <37 weeks of gestation) and low birthweight (LBW, <2.5 kg) [14,15,16,17,18]. This is not only due to higher multiple pregnancy rates, but also ART singletons that are known to be of increased risk of perinatal complications including PTB and LBW [14]. According to a meta-analysis by [19], the risk for PTB after excluding frozen embryo transfer cycles was 10.9% for IVF/ICSI and 6.4% for babies spontaneously conceived.

In 2014, we reported the birth of eleven healthy babies as a result of fresh (FRET) and frozen embryo transfer (FET) conceived with SCS [12,20].Recently, we published the perinatal data of 208 babies born after using SCS. The results were reassuring, if not better than expected [21]. In the present study, we compare the prevalence of the LBW and PTB of 176 singleton babies born after the transfer of SCS embryos between 2013 and 2020 with perinatal outcome results of all IVF babies born in Belgium between 2013 and 2018 (BELRAP data).

Since July 2003, according to the Belgian law, IVF centres are obliged to register all IVF and IVF-related cycles online as part of the Belgian reimbursement policy [22,23,24]. Key indicators covering the most important aspects of IVF treatment are registered by BELRAP (Belgian Register for Assisted Procreation), including the indication for treatment, cycle-specific data (fresh and thawed cycles), data on transfer, complications, early pregnancy, its progression and birth. Data collection is performedvia a remote and secured web-based system. Centres can upload their data and obtainimmediate feedback about missing data, errors, and inconsistencies. Annual reports are published on the website since 2004 (www.belrap.be), and these data are provided to European IVF Monitoring (EIM) and the International Committee for Monitoring Assisted Reproductive Technology (ICMART) since 2002 [25,26].

## 2. Materials and Methods

### 2.1. The Simplified Culture System (SCS)

**Figure 1:**
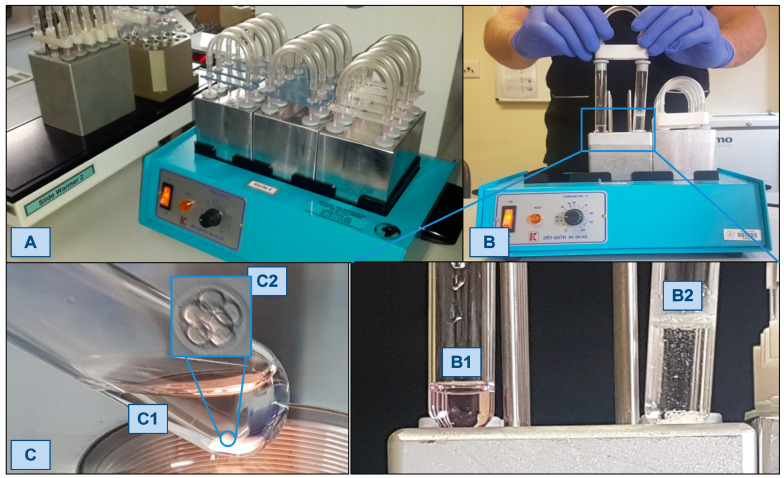
The ‘simplified culture system’ (SCS) performs IVF relaying on a self-contained, air-tight closed environment composed of two standard plain glass medical grade culture tubes connected by catheter tubing (**A**,**B**). One tube serves as a CO_2_ generator through an effervesce reaction between sodium bicarbonate and citric acid releasing sufficient CO_2_ (**B2**), while the other tube contains ‘single-step’ culture medium equilibrating to an atmosphere and pH (between 7.29 and 7.35). With the correct pH, the culture medium shows a pink colour (**B1**,**C1**). With a stereoscopic microscope, the embryo can be observed through the glass wall of the vacutainer (**C2**). The SCS system is consistent with human IVF provided that the tube is kept at 37 °C during fertilization and further embryo culture.

**Figure 2:**
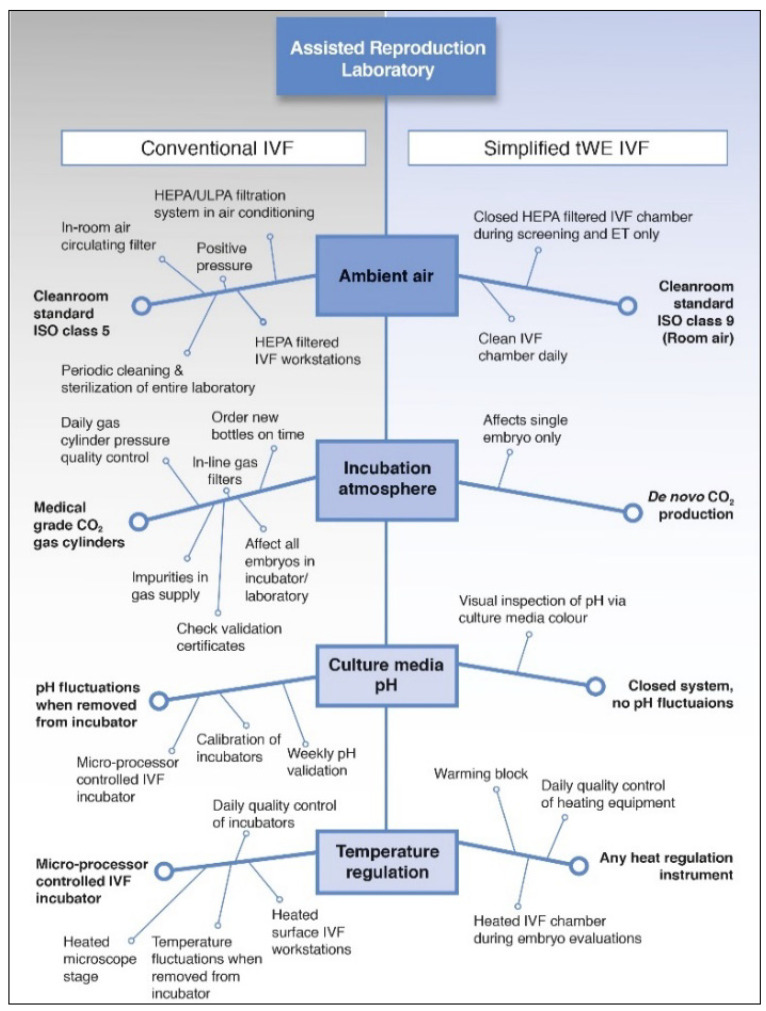
Overview of the differences in essential prerequisites to ensure optimal laboratory quality between a regular IVF and SCS laboratory. The main difference is that in conventional IVF we use incubators with continuous gas regulation, whereas the simplified culture is in a closed tube that is pre-gassed to ensure optimal environment. The simplified culture system therefore only needs to be kept warm and doesnot need a gas supply to be connected to the incubator.

As described before by Van Blerkom et al. [12], our ‘simplified culture system’ (SCS) performs IVF relaying ina self-contained, air-tight closed environment composed of two standard plain glass vacutainers connected by catheter tubing. One tube serves as a CO_2_ generator through an effervesce reaction between sodium bicarbonate and citric acid releasing sufficient CO_2_, while the other tube contains a ‘single-step’ culture medium equilibrating to a defined atmosphere and pH (between 7.29 and 7.35) (Figure 1). The SCS system is consistent with human IVF, provided that the tube is kept at 37 °C during fertilization and the further embryo culture. A summary of the quality control requirements of a regular IVF laboratory versus the SCS laboratory are shown in Figure 2.

### 2.2. Comparative Medical Economics of IVF Systems

We previously studied the dissimilarity in costs when settingup and running a typical conventional high-tech IVF laboratory or a SCS laboratory in order to evaluate how the medical economics of the SCS compares [27]. The investment model we have chosen is the discounted cash flow (DCF) method. The SCS laboratory clearly showed the highest net present value (NPV) being the difference between the present value of cash inflows and the present value of cash outflows over a period of time. Our results showed that the implementation of a SCS laboratory turned out to be the most attractive investment [27].

### 2.3. The SCS Patient Cohort: Materials and Methods

All patients are part of a prospective study performed at the ZOL Hospitals in Genk, Belgium. The methodology of this study was previously described [12,13]. To be selected for IVF, infertile couples were evaluated according to the standard protocol of our centre. Only couples trying to conceive out of any contraceptive plan for at least one year without success were eligible for treatment. Female patients were subjected to a diagnostic work-up including medical history, physical examination, pelvic ultrasound, serum hormone assays, and a hysterosalpingography (HSG), hysterosalpingo-foam sonography (HyFoSy) or hysteroscopy. Laparoscopy was performed in case of suspected tubal pathology, endometriosis, or the presence of an ovarian cyst(s) on ultrasound. For men, at least two semen analyses were performed prior to treatment according to the WHO guidelines [28,29].

All women were less than 43 years old, presented a minimum of sixoocytes at collection, suffered from tubal occlusion, mild-to-moderate endometriosis, or unexplained infertility. Couples with mild-to-moderate male infertility were included provided the number of motile spermatozoa after processing (IMC or Inseminating Motile Count) was above 1 million. For all patients with open tubes, at least threeor fourintrauterine inseminations (IUI) were performed before starting IVF or ICSI, provided a severe male factor could be excluded (IMC < 1 million).

For ovarian stimulation, oocytecollection, and semen processing, we followed the Genk protocols as described previously [13].

Embryo transfer was performed 3–5 days after oocyte aspiration. For luteal phase supplementation, we used 600 mg micronized progesteronein three separate dosages starting the evening of oocyte retrieval and continuing until 18 days after ovum pick-up. Progesterone supplementation was continued when the pregnancy test was positive until the day of ultrasound 5–6 weeks after oocyte retrieval [13].

Surplus embryos were vitrified using a commercially available vitrification kitaccording to the manufacturer’s protocol.

### 2.4. The SCS Patient Cohort: Outcome Parameters

All babies born after the transfer of SCS fresh and cryo/thawed embryos were prospectively studied. Three months after the expected day of delivery patients received a phone call by a dedicated midwife to inquire about obstetrical and perinatal outcome with special attention to the date of delivery, mode of delivery, gender of the baby, birthweight, and the presence of congenital malformations. The data obtained were always compared with the information we received from the obstetrical unit where the delivery took place.

### 2.5. Comparison with BELRAP Data

Due to our very low multiple pregnancy rate (11 twin pregnancies or 5.8%, 22 babies) only SCS singletons were statistically compared between both groups. PTB (<37 weeks) and LBW (<2.5 kg), representing the leading causes for perinatal and infant morbidity and mortality, were compared between 105 singleton babies born fresh ET using our simplified IVF method (SCS) and 16 288 IVF/ICSI singletons reported in the Belgian register for assisted procreation (BELRAP) born after a fresh ET between 2013 and 2018. All cycles in which SCS was performed were removed in the BELRAP registry.

Subsequently our data of 105 SCS babies werecompared with the BELRAP group, in which only IVF was performed. Subsequently, we adjusted for (i) age of the mother, (ii) day of transfer, (iii) pituitary inhibition protocol, (iv) rank of cycles, (v) number of oocytes retrieved, (vi) number of embryos transferred, and (vii) gender of the baby.

We also compared PTB and LBW values of our 71 SCS frozen ETbabies with the PTB and LBW rate of 11,418 IVF/ICSI babies born after frozen ET from the BELRAP registry during the same study period. To compare both groups after adjusting for the different variables mentioned above was impossible because the registration of babies born after frozen ET in the Belgian registry didnot mention whether IVF and/or ICSI wereused in the original cycle.

### 2.6. Ethical Committee Approval

The Ethical Committees of Genk and the Free University of Brussels approved the ongoing prospective study registered as B.U.N. 143201110348. All patients signed an informed consent. The study comparing the perinatal outcome parameters of 176 SCS singletons and all IVF singletons in Belgium during the period 2013–2018 was approved by the ethical Committee of Genk (Internal reference number 19/0047R). A data transfer agreement for academic research purpose only was signed between the Belgian College of Physicians for Reproductive Medicine and the ZOL Hospitals, Genk, on 15 April 2021.

### 2.7. Statistics

First of all, PTB and LBW rates for all singletons born after fresh ET and frozen ET after using SCS are compared with the outcomes of all IVF/ICSI babies reported in the Belgian Register for Assisted Procreation (BELRAP) by using the Fisher’s exact test.

Secondly, we compared all SCS singletons after fresh transfer (*n* = 105) with 2695 babies from the BELRAP registry born after “IVF only” (no ICSI). Information about premature gestational age at delivery (<37 weeks), low birthweight (<2.5 kg), age of the mother (<25, 25–29, 30–34, 35–39, ≥40 year),day of transfer (2–3, 4–5 days), pituitary inhibition protocol (agonist–long, agonist–short, antagonist), rank of cycles (1, 2, 3, >3), number of oocytes retrieved (<6, 6–10, 11–15, 16–20, >20),number of embryos transferred (1, 2, >2),and gender of these babies was available.

A logistic regression model, with a Firth correction, is employed to compare the IVF-only and SCS fresh pregnancies for the two binary endpoints, i.e., premature gestational age at delivery (<37 weeks, ≥37 weeks) and low birthweight (<2.5 kg, ≥2.5 kg).The Odds Ratio for SCS versus IVF only is presented with a 95% confidence interval.An adjusted estimate for the Odds Ratio is obtained by extending the logistic regression model with the following risk factors:age of the mother, day of transfer, pituitary inhibition protocol, rank of cycles, number of oocytes retrieved, number of embryos transferred and gender of the baby.A 5% level of significance is used. The statistical analyses were performed with SAS Version 9.4 (SAS Institute, Cary, NC, USA).

## 3. Results

A total of 3.8% of singletons born after SCS and fresh ET were born before 37 weeks, and2.9% had a low birthweight (<2.5 kg). For all IVF/ICSI cycles (BELRAP), 9.2% of the babies were delivered before 37 weeks, and 8.4% hadbirthweights below 2.5 kg. When comparing all 16,288 IVF/ICSI (BELRAP) with 105 fresh ET SCS singletons, we found a significant higher prevalence of LBW in the BELRAP group (*p* = 0.03) (Table 1).

With respect to our frozen ET singletons, the PTB rate was 8.4% (6/71) for SCS and 10.1% (1154/11,418) for the BELRAP group (*p* = 0.64), witha LBW rate of 4.2% (3/71) for SCS and 5.2% (573/11,087) for BELRAP group (*p* = 0.72) (Table 2).

In 2695 out of 16,288 IVF/ICSI registered BELRAP cases, only IVF was performed. In the large majority of cycles, ICSI or a combination of IVF and ICSI was performed. When examining the “IVF-only” BELRAP group, 10.2% of the babies were born before 37 weeks and 9.8% with a birthweight below 2.5 kg. When compared to our 105 SCS singletons, PTB and LBW were significantly higher in the BELRAP group (PTB: OR 2.852, 95% CI 1.042–7.803, *p*-value 0.0413; LBW: OR 3.692, 95% CI [1.163–11.721], *p*-value 0.0267) (Table 3 and Table 4).

The “IVF-only” and SCS pregnancies differ with respect to pituitary inhibition (*p* = 0.0001), day oftransfer (*p* = 0.0001), number of eggs retrieved (*p* = 0.0001), and number of embryos transferred (*p* = 0.0029) (Table 5).Via a logistic regression model (with a Firth correction), risk estimates for LBW and PTB were adjusted for the seven risk factors mentioned above. After adjustment, the odds for prematurity (OR = 2.627, 95% CI [1.013; 6.816], *p* value 0.0471) and low birth weight (OR = 3.267, 95% CI [1.118; 9.549], *p* value 0.0305) remained significantly higher for the BELRAP babies (Table 4).

For SCS, 76.2% of the procedures used an antagonist stimulation protocol, 68.6% of the embryos were transferred at days 4–5, and in 87.6%, one embryo was transferred. For the “IVF-only” BELRAP singletons, the antagonist protocol was only used in 47% of cases, 35% of the embryos were transferred on days 4–5, and in 73.2% of cases, a single embryo transfer was performed (Table 5).

We observed no statistically significant differences between SCS and BELRAP records for the age of the mother, rank of the fresh cycle, and gender of the baby (Table 5).

General characteristics for patients delivering singletons after SCS in fresh and frozen ET cycles are shown in Supplemental Table S1. The perinatal and obstetric outcome results for patients delivering singletons after SCS in fresh and frozen ET cycles are shown in Supplemental Table S2.

One perinatal mortality was reported in the fresh ET group: a babyborn at 28 weeks gestation after abruptio placentae died immediately after birth. The reason for the abruptio remained unclear. One congenital malformation in the SCS frozen ET group was recorded: a clubfoot.

## 4. Discussion

Infertility is a universal health issue and the large majority of childless couples are residents of LMIC. Lowering the costs associated with IVF laboratory procedures is urgently required for infertility treatment to become integrated into mainstream reproductive healthcare in LMIC. We previously reported thatuse of a simplified and inexpensive method of culture for IVFyields outcomeresults in terms of pregnancy rate per cycle that are similar to those using current, conventionalhigh-costIVF [12], or ICSI in a selected patient cohort [13]. While using a new innovative method of assisted reproduction, data on safety are extremely important. To rule out suggestions that this novel technology does not exert harmful effects on embryo development, reassuring perinatal outcome results are required before promoting this technique on a global scale. In this regard, the actual literature dealing with perinatal outcome following IVF and IVF-related procedures, shows for nearly all studies that aworse perinatal outcome occurs after IVF/ICSI when compared to natural conception and non-IVF medically assisted reproduction [16,17,18,19,30,31,32]. Therefore we prospectively examined the perinatal outcome of our SCS babies with special attention to PTB and low LBW, the leading causes of perinatal and infant morbidity and mortality [33]. Because of a very low number of twin babies [22], only SCS singletons were statistically investigated in this study.

We found an unanticipated low prevalence of PTB and LBW for singletons after fresh ET, significantly lower when compared to published data dealing with conventional IVF and/or ICSI worldwide [16,17,18,30,31,32].

We first compared PTB and LBW between fresh ET SCS and Belgian conventional IVF/ICSI singletons, making use of the BELRAP data. The LBW rate was significantly lower in the SCS group (Table 1). Secondly, we compared the SCS singletons with singletons born after regular IVF only and excluded those that were ICSI only or a combination of IVF and ICSI for the simple reason that severe male infertility cases were not treated with SCS; consequently, their inclusion could introduce a potential bias that can be responsible for different perinatal outcomes. Although the reports in the literature are contradictory, ICSI technology itself might influence perinatal outcome as well [34,35]. We also corrected for another seven confounding factors as mentioned before.

Surprisingly, in as low as 17% of our registered Belgian ART procedures performed during the study period, only IVF has been performedcompared to 29% in our centre. Our data indicate that ICSI is performed much too often in Belgium and elsewhere without good evidence for its necessity [36,37,38]. Nevertheless, after correction for all these confounding factors, we still observed a significant lower PTB and LBW in the SCS group.

Different factors can be forwarded to explain this finding. First, in most cases, single embryotransfer was performed (87.6%), and because an ultrasound was always performedbetween 7 and 9 weeks of gestation, we were able to exclude vanishing twins in the SCS Group, which are associated with a worse perinatal outcome [39,40,41]. In the BELRAP group, becausesingle embryo transfers were performedin 73.2% of cycles, it was impossible to rule out vanishing twins in this group because this item was not registered.

Second, because SCS is performed in an enclosed system with temperature control as the only variable, we avoid as much as possible perturbations in temperature, as well as any transient changes in CO_2_ and oxygen concentration and light that can be caused by repeated periods of conventional morphological assessment of embryos incubated in standard incubators. If it can be assumed that our enclosed environment more closely approximates natural conception conditions, rather than those associated with microdops under oil or open culture with larger medium volumes using specialized culture dishes, epigenetic modifications that are iatrogenic in origin may be less likelyto occur(see below).Animal models show convincing evidence that epigenetic changes in genes involved in growth and development are occurring during the IVF process that subsequently alter foetal phenotype and long-term health [42]. Epigenetic reprogramming is potentially susceptible to multiple environmental and iatrogenic influences occurring prior to fertilization (e.g., mode or ovarian stimulation, gamete cryopreservation) and after fertilization during the preimplantation stages in vitro(e.g., pH, metabolism-associated changes in medium composition, fluctuations in temperature, oxygen/CO_2_ tension, light exposure) [43].

Perinatal outcomes after using time-lapse monitoring systems that incorporatereduced embryo handling and consistent environmental conditions also showed higher mean birthweight and better prematurity and LBW rates after fresh and frozen embryo transfer when compared to regular IVF; however, as reported here, the benefit in these studies is less pronounced compared to use of the SCS [44,45]. Prospective human data are needed to elucidate exogenous outcome, and if so, the mechanisms involved; in this context, we suggest that special attention should be given to comparing different IVF culture systems such as the SCS with theirs [46].

It has previously been reported that different culture media used for the in vitroculture of human embryos can affect birthweight of live-born singletons [47,48]. In our study, Irvine was the medium of choice for SCS, but for all babies born after IVF in the BELRAP registry, the culture medium used was not reported. However, it is worth noting that it is very unlikely that the use of different media explains our significantly lower PTB rate infresh ET SCS singletons since PTB has never been observed to be influenced by differences in culture media.

When examining the babies born after frozen ET, we observed similar rates of PTB and LBW in the SCS and BELRAP group. The possible benefit of an enclosed system in fresh cycles seems to decrease or even disappear when cryopreservation is used. Cryopreservation has been reported as one of the mechanisms responsible for epigenetic modifications in human embryos [14], and several reports have shown that singletons born after frozen ET have a higher birthweight and are of increased risk of large-for-gestational-age (LGA), but are less likely to be LBW [49,50]. This finding is supported by comparisons to spontaneous pregnancies, where those naturally conceived do not show the same incidence of LBW as seen with FET.On the other hand, babies born after both fresh ET and frozen ET are associated with PTB [51,52]. UnlikeLBW, preterm birth is not seen less often after frozen ET when compared to fresh ET.

The major strength of this study is that all data were registered prospectively. The follow-up of the SCS pregnancies was meticulous and reliable. Whenever needed, our team contacted patients again to clarify any inaccuracies. BELRAP data are also considered reliable since the online registration is always started the first day of the IVF treatment cycle via a secured web-based system with regular feedback about missing data, errors, and inconsistencies.

It is important to consider that our study has some limitations as well. First, this study might have been underpowered to show a difference in perinatal outcome between groups. Given that most pregnancy complications occur at an incidence of 10% or lower, one would not expect a difference in these small study numbers. Moreover, sample-size calculations were not performed for an equivalence or non-inferiority hypothesis, which would have required a huge sample, which was impossible to achieve in our setting.

Secondly, our main outcome perinatal parameters were PTB and LBW because they are the leading causes for perinatal and infant morbidity and mortality and the most reliable to use when comparing BELRAP and our data. Although we adjusted for eight parameters when comparing SCS and BELRAP singletons, residual confounding cannot be excluded related to duration and cause of infertility that we couldnot adjust for due to the lack of reliable data in the Belgian registration. The same counts for other characteristics and variables influencing perinatal outcome such as the smoking habits of both partners, BMI, parity of women, socio-cultural background, race and pregnancy-related hypertensive disorders, and PROM (premature rupture of membranes).

Thirdly, although very unlikely, there also might be a small difference in our patient cohort compared to the BELRAP group because our SCS pregnancies were generated from a single laboratory and clinic following strict standard protocols for diagnosis and selection of patients for different treatment options, and these protocols are not always the same in all IVF centres.

Being aware of these limitations, we still must realize that, for singletons born after SCS in a fresh cycle, we observed a low PTB and LBW, obtaining figures similar to those published onsingletons born after natural conception.

## 5. Conclusions

Our data show that SCS can be used as a safe alternative for regular ART with very promising perinatal outcome results. Provided the results of this study can be reproduced in other centres, SCS can be used safely with no detrimental effects on perinatal outcome. Because of the low costs associated with SCS, this new method opens up perspectives to make assisted reproductive techniques available to a much larger part of the worlds’ infertile population. This can be regarded as an important breakthrough in terms of human rights, equity, and social justice that has been much discussed by international organizations, for infertile couples in general and those inLMIC in particular requiring advanced fertility treatment needing IVF. We believe that our SCS findings offer the needed outcome results for the different stakeholders to support its application where they have stated publicly such a system is needed.

## Figures and Tables

**Table 1:** Preterm birth and low birthweight comparison between SCS and all IVF/ICSI BELRAP singletons, after fresh embryo transfer—number (%).

		All IVF/ICSI (BELRAP)	SCS	*p* Value (Fisher’s Exact Test)
Gestational age at delivery				0.0549
	<37 weeks	1505 (9.2)	4 (3.8)	
	≥37 weeks	14,783 (91.8)	101 (96.2)	
Birthweight				0.0399
	< 2.5 kg	1338 (8.4)	3 (2.9)	
	≥2.5 kg	14,521 (91.6)	102 (97.1)	

**Table 2:** Preterm birth and low birthweight comparison between SCS and all IVF/ICSI BELRAP singletons after frozen embryo transfer—number (%).

		All IVF/ICSI (BELRAP)	SCS	*p* Value (Fisher’s Exact Test)
Gestational age at delivery				0.6454
	<37 weeks	1154 (10.1)	6 (8.4)	
	≥37 weeks	10,264 (89.9)	65 (91.6)	
Birthweight				0.7210
	<2.5 kg	573 (5.2)	3 (4.2)	
	≥2.5 kg	10,514 (94.8)	68 (95.8)	

**Table 3:** Preterm birth and low birthweight comparison between SCS and BELRAP “IVF-only” singletons born after fresh ET, before adjustment—number (%).

		IVF Only (BELRAP)	SCS	*p* Value (Fisher’s Exact Test)
Gestational age at delivery				0.0296
	<37 weeks	272 (10.2)	4 (3.8)	
	≥37 weeks	2406 (89.8)	101 (96.2)	
Birthweight				0.0161
	<2.5 kg	264 (9.8)	3 (2.9)	
	≥2.5 kg	2431 (90.2)	102 (97.1)	

**Table 4:** Logistic regression models for preterm birth and low birthweightoutcomes comparing SCS and BELRAP “IVF-only” singletons after fresh embryo transfer (OR = Odds Ratio, CI = Confidence Interval).

	OR (95% CI) BELRAP vs. SCS	*p* Value (Logistic Model)
Gestational age at delivery < 37 w		
No correction for risk factors	2.852 [1.042; 7.803]	0.0413
Correction for risk factors	2.627 [1.013; 6.816]	0.0471
Birth weight < 2.5 kg		
No correction for risk factors Correction for risk factors	3.692 [1.163; 11.721] 3.267 [1.118; 9.549]	0.0267 0.0305

**Table 5:** Descriptive statistics of the risk factors for which the adjustment was performed—number (%) fresh ET group.

		IVF only (BELRAP)	SCS	*p* Value
Age of mother (years)				0.7615
	<25	64 (2.4)	3 (2.9)	
	25–29	539 (20.0)	21 (20.0)	
	30–34	1116 (41.4)	42 (40.0)	
	35–39	742 (27.5)	33 (31.4)	
	≥40	234 (8.7)	6 (5.7)	
Day of transfer				0.0001
	2–3	1751 (65.0)	33 (31.4)	
	4–5	943 (35.0)	72 (68.6)	
Pituitary inhibition				0.0001
	Agonist long	1016 (37.8)	14 (13.3)	
	Agonist short	408 (15.2)	11 (10.5)	
	Antagonist	1265 (47.0)	80 (76.2)	
Rank of fresh cycle				0.1691
	1	1445 (53.7)	49 (46.7)	
	2	696 (25.9)	30 (28.6)	
	3	292 (10.9)	18 (17.1)	
	>3	257 (9.6)	8 (7.6)	
Number of oocytes retrieved				0.0001
	<6	761 (28.2)	4 (3.8)	
	6–10	1083 (40.2)	50 (47.6)	
	11–15	609 (22.6)	36 (34.3)	
	16–20	190 (7.1)	11 (10.5)	
	>20	52 (1.9)	4 (3.8)	
Number of embryos transferred				0.0029
	1	1970 (73.2)	92 (87.6)	
	2	650 (24.1)	12 (11.4)	
	≥3	73 (2.7)	1 (1.0)	
Gender of baby				0.1347
	Male	1429 (53.3)	64 (61.0)	
	Female	1254 (46.7)	41 (39.0)	

## Data Availability

All data generated or analyzed in this study are included in this published article.

## Supplementary Materials

The following supporting information can be downloaded at: https://www.mdpi.com/article/10.3390/jcm12062264/s1, Table S1. General characteristics for patients delivering singletons after SCS in fresh (FRET) and frozen (FET) embryo transfer cycles (SET = single embryo transfer, DET = double embryo transfer); Table S2. Perinatal and obstetric outcome results for patients delivering singletons after SCS in fresh (FRET) and frozen (FET) embryo transfer cycles (SGA = small for gestational age; LGA = large for gestational age).
