# Supplementary material for: Promising Perinatal Outcome after Using a Simplified Low-Cost IVF Culture System Specifically Designed for Resource-Poor Countries
Source: J Clin Med. 2023 Mar 15;12(6):2264. doi: 10.3390/jcm12062264 (PMC10059708; doi:10.3390/jcm12062264)
Supplement: Supplementary file 1 [file jcm-12-02264-s001.zip › jcm-2260131-supplementary.pdf]

Suppl. Table S1. General characteristics for patients delivering singletons after SCS in fresh (FRET) and frozen (FET) embryo transfer cycles (SET = single embryo transfer, DET = double embryo transfer).

	**SCS-FRET**			**SCS-FET**	
	**n = 105**	%		**n = 71**	%
**Female age (mean +/- SD)**	33 +/- 5,0			31,2 +/- 4,2	
**BMI (kg/m2) (mean +/- SD)**	24,7 +/- 3,0			24,9 +/- 3,0	
**Primary infertility**	62	59,1		37	52,1
**Secondary infertility**	43	41		34	47,9
**Smokers**	12	11,4		8	11,3
**Rank (cycle)**					
**1**	49	46,7		36	50,7
**2 or 3**	48	45,7		18	25,4
**>3**	8	7,6		17	23,9
**Ovarian stimulation**					
**Antagonist**	92	87,6	**Natural cycle**	54	76,1
**Short Agonist**	10	9,5	**Substitution**	17	23,9
**Long Agonist**	3	2,9			
**Day Embryo Transfer**					
**Day 2-3**	33	31,4			
**Day 4-5**	72	68,6			
**Embryos transferred**					
**SET**	92	87,6		62	87,3
**DET**	13	12,4		9	12,7

Suppl. Table S2. Perinatal and obstetric outcome results for patients delivering singletons after SCS in fresh (FRET) and frozen (FET) embryo transfer cycles (SGA = small for gestational age; LGA = large for gestational age).

	**SCS-FRET**		**SCS-FET**	
	**n = 105**	%	**n = 71**	%
**Birthweight (mean +/- SD)**	3360 +/- 554		3478 +/- 560	
**Duration pregnancy (mean +/- SD)**	39,4 +/- 2,5		39,3 +/- 1,7	
**<32 weeks**	2	1,9	1	1,4
**< 37 weeks**	4	3,8	6	8,5
**< 1,5 kg**	1	1	1	1,4
**< 2,5 kg**	3	2,9	3	4,2
**> 4,2 kg**	7	6,7	6	8,4
**SGA (< P10)**	15	14,3	7	9,9
**LGA (> P90)**	11	10,5	11	15,5
**Perinatal mortality**	1	1	0	0
**Congenital malformation**	0	0	1	1,4
**Delivery (%)**				
**Vaginal**	78	74,3	49	69
**Vacuum Extraction**	5	4,8	5	7
**CesareanSection**	22	21	17	23,9
